# Supplementary material for: A predictive screening tool to detect diabetic retinopathy or macular edema in primary health care: construction, validation and implementation on a mobile application
Source: PeerJ. 2015 Nov 24;3:e1404. doi: 10.7717/peerj.1404 (PMC4662592; doi:10.7717/peerj.1404)
Supplement: Note S1 [file peerj-03-1404-s002.doc]

APPENDIX

The mobile app is available in the following stores:

- Play Store (Android).

- App Store (iPhone).

The name of this app is *Diabetic retinopathy predictor* and it is free for all users of these operating systems.
